# The chromosomal genome sequence of the lesser starlet coral, *Siderastrea radians* (Pallas, 1766) (Scleractinia: Rhizangiidae) and its associated microbial metagenome sequences

**DOI:** 10.12688/wellcomeopenres.27286.1

**Published:** 2026-07-30

**Authors:** Camile Avelino, Richard Karp, Andrew Baker, Sebastian Metz, Michael Sweet, Graeme Oatley, Elizabeth Sinclair, Eerik Aunin, Noah Gettle, Camilla Santos, Michael Paulini, Haoyu Niu, Victoria McKenna, Rebecca O’Brien, Wellcome Sanger Institute Tree of Life Management, Samples and Laboratory team, Wellcome Sanger Institute Scientific Operations: Sequencing Operations, Wellcome Sanger Institute Tree of Life Core Informatics team, EBI Aquatic Symbiosis Genomics Data Portal Team, Aquatic Symbiosis Genomics Project Leadership

**Affiliations:** 1 Aquatic Research Facility, Nature-based Solutions Research Centre, University of Derby, Derby, England, UK; 2 Department of Marine Biology and Ecology, University of Miami, Miami, Florida, USA; 3 Tree of Life Programme, Wellcome Sanger Institute, Hinxton, England, UK

**Keywords:** Siderastrea radians, lesser starlet coral, genome sequence, chromosomal, Scleractinia, microbial metagenome assembly

## Abstract

We present a genome assembly from a specimen of *Siderastrea radians* (lesser starlet coral; Cnidaria; Anthozoa; Scleractinia; Rhizangiidae). The genome sequence has a total length of 807.19 megabases. Most of the assembly (94.17%) is scaffolded into 14 chromosomal pseudomolecules. The mitochondrial genome has also been assembled, with a length of 19.38 kilobases. Gene annotation of this assembly by Ensembl identified 47 051 protein-coding genes. From the metagenome data, we recovered two binned metagenomes assigned to the bacterial phylum Bacteroidota and class Bacteroidia.

## Species taxonomy

Eukaryota; Opisthokonta; Metazoa; Eumetazoa; Cnidaria; Anthozoa; Hexacorallia; Scleractinia; Refertina; Rhizangiidae; *Siderastrea*; *Siderastrea radians* (Pallas, 1766) (NCBI:txid214988).

## Background

*Siderastrea radians* (Pallas, 1766), commonly known as the lesser starlet coral, plays a pivotal role in the ecology of Caribbean coral reefs. This species predominantly inhabits shallow-water environments, including limestone pavements, seagrass beds, nearshore rubble, and coral reefs, where it can form dense monospecific colonies that cover up to 90% of the substrate ( Vermeij, 2005).

Geographically, *S. radians* can be found throughout the Tropical Atlantic, extending from the Caribbean islands to regions along the Brazilian coast ( Evangelista *et al.*, 2018). There is considerable overlap in the distribution of species belonging to this genus, which does mean that *in-situ* identification between closely related species ( *S. radians* and *S. siderea* for example) can be challenging. In general, *S. radians* forms small encrusting colonies (<300 mm), characterised by smaller corallites when compared to related species like *S. siderea.* The cerioid structure of its corallites manifests in rounded, irregular distributions, typically measuring between 2.5 to 3 mm in diameter ( Sadler *et al.*, 2014). Additionally, it typically hosts the algal symbiont type *Breviolum* (B5a) ( LaJeunesse, 2002).

*S. radians* is classified as a ‘weedy coral’, able to tolerate significant stressors and is currently listed as Least Concern on the International Union for Conservation of Nature (IUCN) Red List ( Rodríguez-Martínez *et al.*, 2022). For example, it appears to be resilient to thermal fluctuations and can tolerate hyper-saline conditions, indicative of its broader ecological tolerance compared to other coral species ( Sadler *et al.*, 2014; van der Merwe *et al.*, 2014). The weedy nature also refers to its ability to mature rapidly and reproduce effectively through brooding i.e. fertilisation occurs internally, and planulae are released from the parent colony. This strategy enhances local recruitment success as planulae tend to settle nearby the adults ( Speare *et al.*, 2020; Takahashi *et al.*, 2008), and as such *S. radians* has a marked density-dependent settlement dynamic ( Vermeij & Sandin, 2008). This species therefore often exhibits rapid recovery and colonisation of disturbed habitats ( Speare *et al.*, 2020).

## Methods

### Sample acquisition

**Figure 1.**
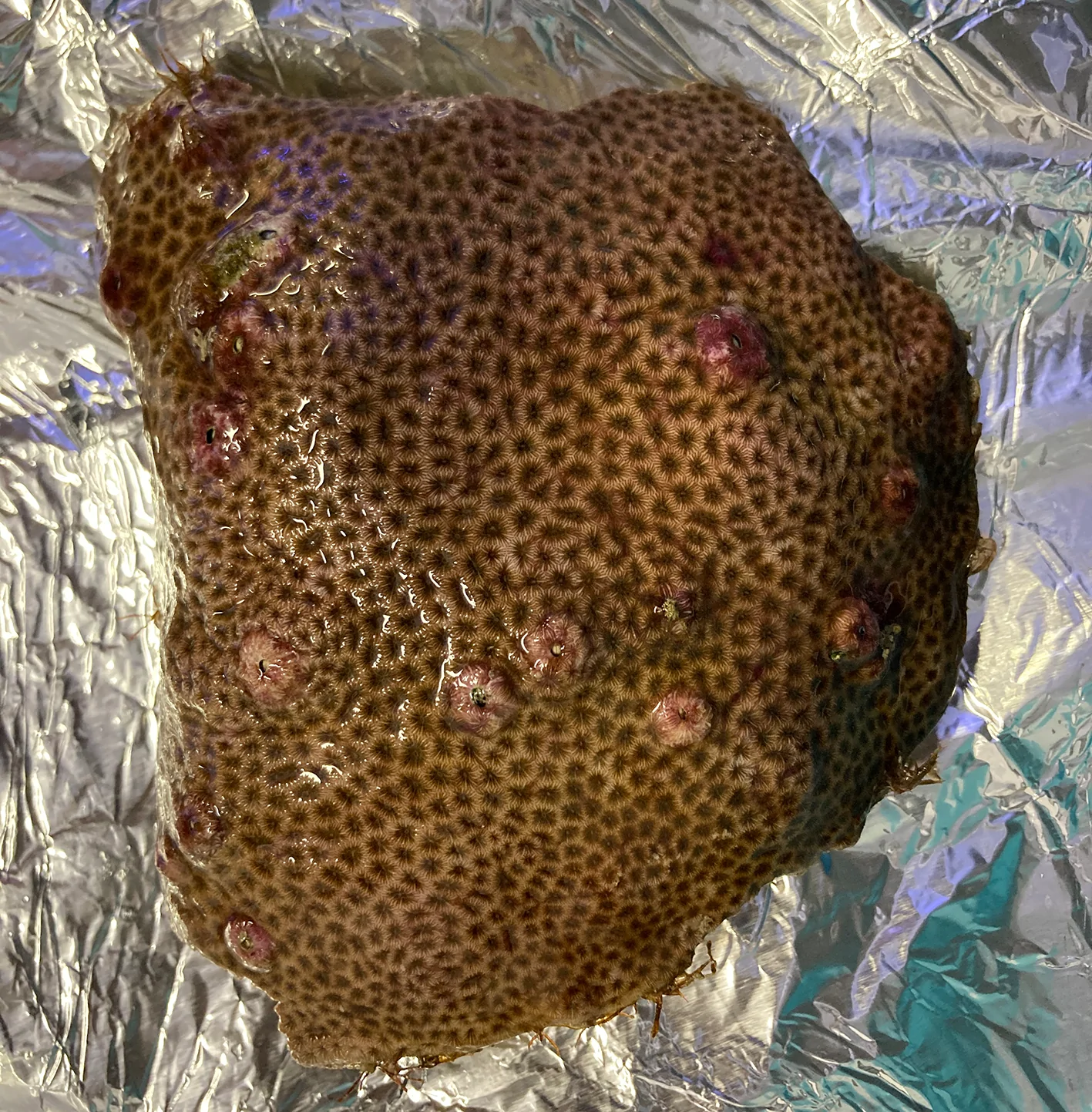
Photograph of the *Siderastrea radians* (jaSidRadi1) specimen used for genome sequencing.

A specimen of *S. radians* (specimen ID UDUK0000164, ToLID jaSidRadi1; Figure 1) was visually identified and collected from nearshore boulders at a depth of 2 m, adjacent to seawalls in Miami, Florida, USA (latitude 25.677, longitude −80.1654) using a hammer and chisel in March 2021 under permit FWC SAL-21-1182-SRP. The specimen was transported to the University of Miami, and 10 samples were taken from the colony on 2021-03-30. These samples were placed in an Eppendorf tube and immediately flash frozen at −80 °C. The jaSidRadi1 specimen was also used for RNA sequencing.

### Nucleic acid extraction

Protocols for high molecular weight (HMW) DNA extraction developed at the Wellcome Sanger Institute (WSI) Tree of Life Core Laboratory are available on protocols.io ( Howard *et al.*, 2025). The jaSidRadi1 sample was weighed and triaged to determine the appropriate extraction protocol. Tissue was homogenised by cryogenic disruption using the Covaris cryoPREP ^®^ Automated Dry Pulverizer. HMW DNA was extracted using the Manual MagAttract protocol. DNA was sheared into an average fragment size of 12–20 kb following the Megaruptor®3 for LI PacBio protocol. Sheared DNA was purified by automated SPRI (solid-phase reversible immobilisation). The concentration of the sheared and purified DNA was assessed using a Nanodrop spectrophotometer and Qubit Fluorometer using the Qubit dsDNA High Sensitivity Assay kit. Fragment size distribution was evaluated by running the sample on the FemtoPulse system. For this sample, the final post-shearing DNA had a Qubit concentration of 1.83 ng/μL, with a fragment size of 8.9 kb.

RNA was extracted from tissue of the jaSidRadi1 sample in the Tree of Life Laboratory at the WSI using the RNA Extraction: Automated MagMax™ *mir*Vana protocol. The RNA concentration was assessed using a Nanodrop spectrophotometer and a Qubit Fluorometer using the Qubit RNA Broad-Range Assay kit. Analysis of the integrity of the RNA was done using the Agilent RNA 6000 Pico Kit and Eukaryotic Total RNA assay.

### PacBio HiFi library preparation and sequencing

Library preparation and sequencing were performed at the WSI Scientific Operations core. Libraries were prepared using the SMRTbell Prep Kit 3.0 (Pacific Biosciences, California, USA), following the manufacturer’s instructions. The kit includes reagents for end repair/A-tailing, adapter ligation, post-ligation SMRTbell bead clean-up, and nuclease treatment. Size selection and clean-up were performed using diluted AMPure PB beads (Pacific Biosciences). DNA concentration was quantified using a Qubit Fluorometer v4.0 (ThermoFisher Scientific) and the Qubit 1X dsDNA HS assay kit. Final library fragment size was assessed with the Agilent Femto Pulse Automated Pulsed Field CE Instrument (Agilent Technologies) using the gDNA 55 kb BAC analysis kit.

The sample was sequenced on a Revio instrument (Pacific Biosciences, California, USA). Prepared libraries with a molarity greater than 2 nM were normalised to 2 nM, and 15 μL was used for making complexes. For libraries with a molarity below 2 nM, the available 10 μL library volume was used without normalisation. Primers were annealed and polymerases were bound to generate circularised complexes, following the manufacturer’s instructions. Complexes were purified using 1.2× SMRTbell beads, diluted to the Revio loading concentration of 200–300 pM, and spiked with a Revio sequencing internal control. The sample was sequenced on a Revio 25 M SMRT cell. SMRT Link software (Pacific Biosciences), a web-based workflow manager, was used to configure and monitor the run and to carry out primary and secondary data analysis.

### Hi-C

***Sample preparation and crosslinking***

The Hi-C sample was prepared from 20–50 mg of frozen tissue from the jaSidRadi1 sample using the Arima-HiC v2 kit (Arima Genomics). Following the manufacturer’s instructions, tissue was fixed and DNA crosslinked using TC buffer to a final formaldehyde concentration of 2%. The tissue was homogenised using the Diagnocine Power Masher-II. Crosslinked DNA was digested with a restriction enzyme master mix, biotinylated, and ligated. Clean-up was performed with SPRISelect beads before library preparation. DNA concentration was measured with the Qubit Fluorometer (Thermo Fisher Scientific) and Qubit HS Assay Kit. The biotinylation percentage was estimated using the Arima-HiC v2 QC beads.

***Hi-C library preparation and sequencing***

Biotinylated DNA constructs were fragmented using a Covaris E220 sonicator and size selected to 400–600 bp using SPRISelect beads. DNA was enriched with Arima-HiC v2 kit Enrichment beads. End repair, A-tailing, and adapter ligation were carried out with the NEBNext Ultra II DNA Library Prep Kit (New England Biolabs), following a modified protocol where library preparation occurs while DNA remains bound to the Enrichment beads. Library amplification was performed using KAPA HiFi HotStart mix and a custom Unique Dual Index (UDI) barcode set (Integrated DNA Technologies). Depending on sample concentration and biotinylation percentage determined at the crosslinking stage, libraries were amplified with 10–16 PCR cycles. Post-PCR clean-up was performed with SPRISelect beads. Libraries were quantified using the AccuClear Ultra High Sensitivity dsDNA Standards Assay Kit (Biotium) and a FLUOstar Omega plate reader (BMG Labtech).

Prior to sequencing, libraries were normalised to 10 ng/μL. Normalised libraries were quantified again to create equimolar and/or weighted 2.8 nM pools. Pool concentrations were checked using the Agilent 4200 TapeStation (Agilent) with High Sensitivity D500 reagents before sequencing. Sequencing was performed using paired-end 150 bp reads on the Illumina NovaSeq 6000.

### RNA library preparation and sequencing

Libraries were prepared using the NEBNext® Ultra II Directional RNA Library Prep Kit for Illumina (New England Biolabs), following the manufacturer’s instructions. Poly(A) mRNA was isolated from 100 ng of total RNA using oligo (dT) beads, converted to cDNA, and uniquely indexed; 14 PCR cycles were performed. Libraries were prepared with an intended fragment size of 100–300 bp. Libraries were quantified, normalised, and pooled equimolarly to a final concentration of 2.8 nM before dilution to 150 pM for loading. Sequencing was carried out on the Illumina NovaSeq X, generating paired-end reads.

### Genome assembly

Prior to assembly of the PacBio HiFi reads, a database of *k*-mer counts ( *k* = 31) was generated from the filtered reads using FastK. GenomeScope2 ( Ranallo-Benavidez *et al.*, 2020) was used to analyse the *k*-mer frequency distributions, providing estimates of genome size, heterozygosity, and repeat content.

The HiFi reads were assembled using Hifiasm ( Cheng *et al.*, 2021) with the --primary option. Haplotypic duplications were identified and removed using purge_dups ( Guan *et al.*, 2020). The Hi-C reads ( Rao *et al.*, 2014) were mapped to the primary contigs using bwa-mem2 ( Vasimuddin *et al.*, 2019), and the contigs were scaffolded in YaHS ( Zhou *et al.*, 2023) with the --break option for handling potential misassemblies. The scaffolded assemblies were evaluated using Gfastats ( Formenti *et al.*, 2022), BUSCO ( Manni *et al.*, 2021) and MerquryFK ( Rhie *et al.*, 2020).

The mitochondrial genome was assembled using MitoHiFi ( Uliano-Silva *et al.*, 2023). The MitoHiFi reference was the mitochondrial genome of *Siderastrea radians* (NC_008167.1).

### Assembly curation

The assembly was decontaminated using the Assembly Screen for Cobionts and Contaminants ( ASCC) pipeline. TreeVal was used to generate the flat files and maps for use in curation. Manual curation was conducted primarily in PretextView and HiGlass ( Kerpedjiev *et al.*, 2018). Scaffolds were visually inspected and corrected as described by Howe *et al*. (2021). Manual corrections included 29 breaks, 20 joins, and removal of 40 haplotypic duplications. This reduced the scaffold count by 16.6% and reduced the total assembly length by 5.0%. The curation process is described at sanger-tol/curation-resources . PretextSnapshot was used to generate a Hi-C contact map of the final assembly.

### Assembly quality assessment

The Merqury.FK tool ( Rhie *et al.*, 2020) was run in a Singularity container ( Kurtzer *et al.*, 2017) to evaluate *k*-mer completeness and assembly quality for the primary and alternate haplotypes using the *k*-mer database ( *k* = 31) computed prior to genome assembly. The analysis outputs included assembly QV scores and completeness statistics.

The genome was analysed using the BlobToolKit pipeline, a Nextflow implementation of the earlier Snakemake version ( Challis *et al.*, 2020). PacBio reads were aligned to the assembly using minimap2 ( Li, 2018) and processed with SAMtools ( Danecek *et al.*, 2021) to generate coverage tracks. The pipeline runs BUSCO ( Manni *et al.*, 2021) using lineages identified from the NCBI Taxonomy ( Schoch *et al.*, 2020). For the three basal BUSCO lineages, eukaryota, bacteria and archaea, BUSCO genes are aligned to the UniProt Reference Proteomes database ( Bateman *et al.*, 2023) using DIAMOND BLASTp ( Buchfink *et al.*, 2021). The genome assembly is divided into chunks based on the density of BUSCO genes from the closest taxonomic lineage, and each chunk is aligned to the UniProt Reference Proteomes database using DIAMOND BLASTx. Sequences without DIAMOND BLASTx hits are extracted using seqtk and aligned to the NT database using BLASTn ( Altschul *et al.*, 1990). The outputs are consolidated into a BlobDir for visualisation in BlobToolKit. The BlobToolKit pipeline was developed using nf-core tooling ( Ewels *et al.*, 2020) and MultiQC ( Ewels *et al.*, 2016), with containerisation through Docker ( Merkel, 2014) and Singularity ( Kurtzer *et al.*, 2017).

## Metagenome assembly

The metagenome assembly was generated using MetaMDBG ( Benoit *et al.*, 2024) and binned using MetaBAT2 ( Kang *et al.*, 2019). The resulting bins were optimised and refined using DAS Tool ( Sieber *et al.*, 2018). Prokka ( Seemann, 2014) was used to identify tRNAs and rRNAs in each bin, CheckM ( Parks *et al.*, 2015) (checkM_DB release 2015-01-16) was used to assess bin completeness/contamination, and GTDB-Tk ( Chaumeil *et al.*, 2022) (GTDB release 214) was used to taxonomically classify bins. Taxonomic replicate bins were identified using dRep ( Olm *et al.*, 2017) with default settings (95% ANI threshold). All bins were assessed for quality and categorised as metagenome-assembled genomes (MAGs) if they met the following criteria: contamination ≤5%, presence of 5S, 16S, and 23S rRNA genes, at least 18 unique tRNAs, and either ≥90% completeness or ≥ 50% completeness with fully circularised chromosomes ( Bowers *et al.*, 2017). Bins that did not meet these thresholds, or were identified as taxonomic replicates of MAGs, were retained as ‘binned metagenomes’ provided they had ≥50% completeness and ≤ 10% contamination.

## Genome sequence report

### Sequence data

**Figure 2.**
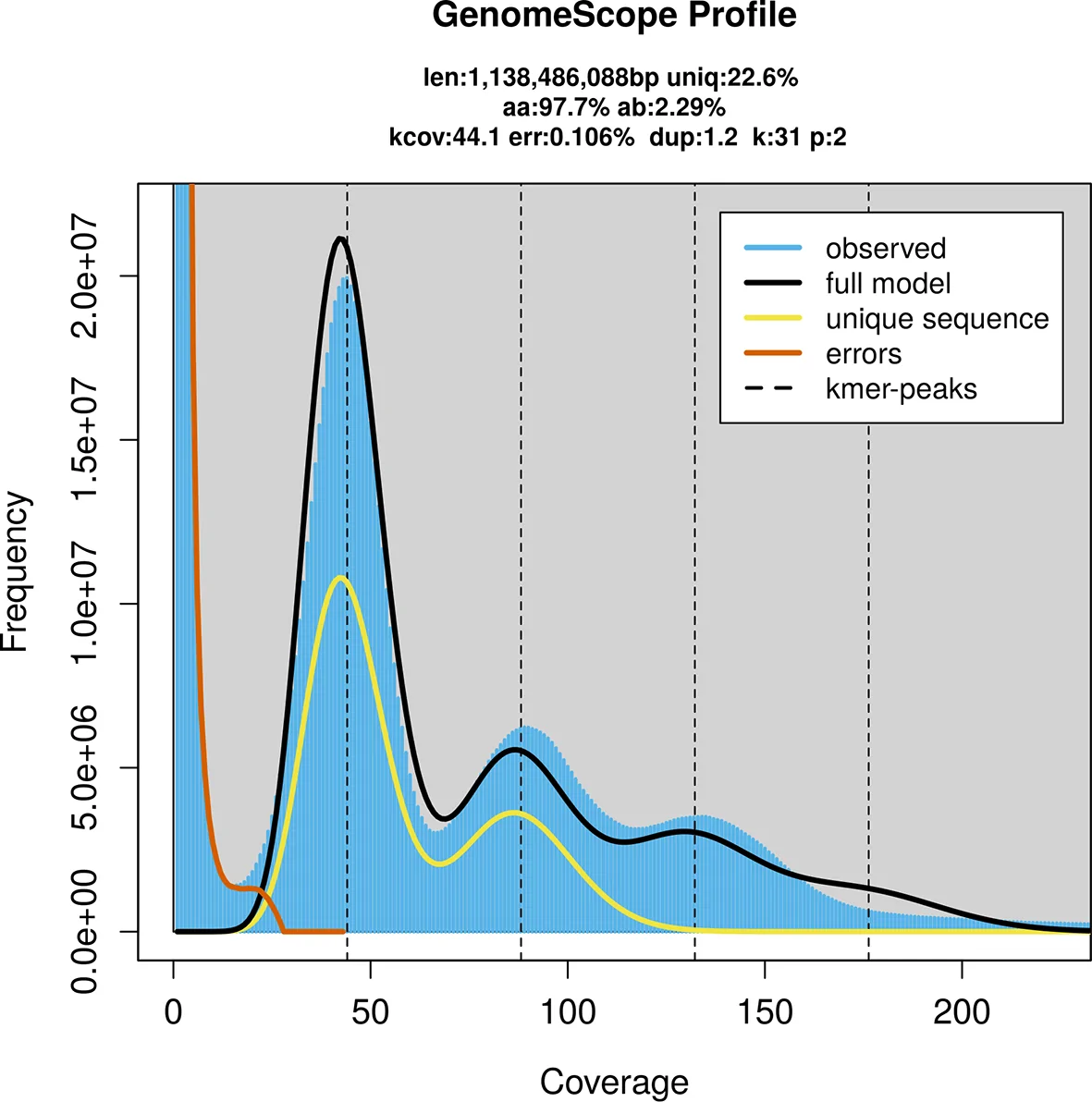
Frequency distribution of *k*-mers generated using GenomeScope2. The plot shows observed and modelled *k*-mer spectra, providing estimates of genome size, heterozygosity, and repeat content based on unassembled sequencing reads.

PacBio sequencing of the *Siderastrea radians* specimen generated 107.00 Gb (gigabases) from 10.31 million reads, which were used to assemble the genome. GenomeScope2.0 analysis estimated the haploid genome size at 1 144.10 Mb, with a heterozygosity of 2.29% and repeat content of 77.42% ( Figure 2). These estimates guided expectations for the assembly. Based on the estimated genome size, the sequencing data provided approximately 88× coverage. Hi-C sequencing produced 80.88 Gb from 267.80 million reads, which were used to scaffold the assembly. RNA sequencing data were also generated and are available in public sequence repositories. Table 1 summarises the specimen and sequencing details.

**Table 1.** Specimen and sequencing data for *Siderastrea radians* (BioProject PRJEB73731).

Platform	PacBio HiFi	Hi-C	RNA-seq
**ToLID**	jaSidRadi1	jaSidRadi1	jaSidRadi1
**Specimen ID**	UDUK0000164	UDUK0000164	UDUK0000164
**BioSample (source individual)**	SAMEA12815080	SAMEA12815080	SAMEA12815080
**BioSample (tissue)**	SAMEA12815107	SAMEA12815100	SAMEA12815106
**Tissue**	modular colony	modular colony	modular colony
**Instrument**	Revio	Illumina NovaSeq 6000	Illumina NovaSeq X
**Run accessions**	ERR12736924; ERR12736925	ERR12737308	ERR13148252
**Read count total**	10.31 million reads	267.80 million read pairs	29.85 million read pairs
**Base count total**	107.00 Gb	80.88 Gb	9.01 Gb

### Assembly statistics

The primary haplotype was assembled, and contigs corresponding to an alternate haplotype were also deposited in INSDC databases. The final assembly has a total length of 807.19 Mb in 538 scaffolds, with 682 gaps, and a scaffold N50 of 56.75 Mb ( Table 2).

**Table 2.** Genome assembly data for *Siderastrea radians.*

Genome assembly	Primary assembly
**Assembly name**	jaSidRadi1.1
**Assembly accession**	GCA_964017195.1
**Alternate haplotype accession**	GCA_964017165.1
**Assembly level**	chromosome
**Span (Mb)**	807.19
**Number of chromosomes**	14
**Number of contigs**	1 220
**Contig N50**	4.19 Mb
**Number of scaffolds**	538
**Scaffold N50**	56.75 Mb
**Organelles**	Mitochondrial genome: 19.38 kb

The scaffold count follows the NCBI assembly statistics and excludes organelle assemblies, which are reported separately where present.

**Figure 3.**
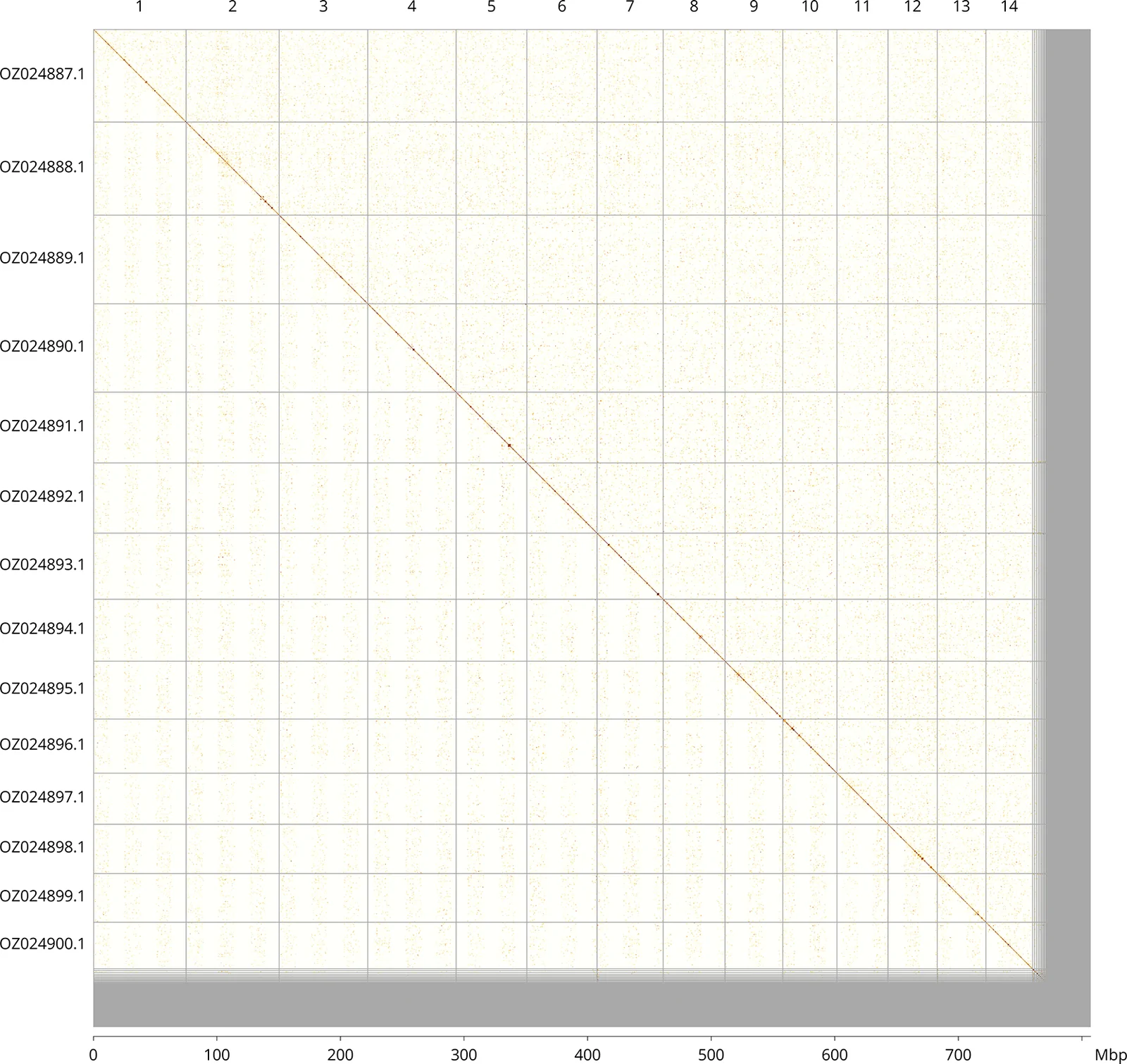
Hi-C contact map of the *Siderastrea radians* genome assembly. Assembled chromosomes are shown in order of size and labelled along the axes, with a megabase scale shown below. The plot was generated using PretextSnapshot.

Most of the assembly sequence (94.17%) was assigned to 14 chromosomal-level scaffolds. These chromosome-level scaffolds, confirmed by Hi-C data, are named according to size ( Figure 3; Table 3).

**Table 3.** Chromosomal pseudomolecules in the primary genome assembly of *Siderastrea radians* jaSidRadi1.

INSDC accession	Molecule	Length (Mb)	GC%
OZ024887.1	1	75.26	39.50
OZ024888.1	2	75.25	39.50
OZ024889.1	3	71.58	40
OZ024890.1	4	71.55	39.50
OZ024891.1	5	57.22	40
OZ024892.1	6	56.75	40
OZ024893.1	7	53.58	40
OZ024894.1	8	50.08	39.50
OZ024895.1	9	46.82	40
OZ024896.1	10	43.74	40
OZ024897.1	11	41.35	40
OZ024898.1	12	39.72	39.50
OZ024899.1	13	39.50	40
OZ024900.1	14	37.71	40

The mitochondrial genome was also assembled (length 19.38 kb, OZ024901.1). This sequence is included as a contig in the multifasta file of the genome submission and as a standalone record.

### Assembly quality metrics

**Figure 4.**
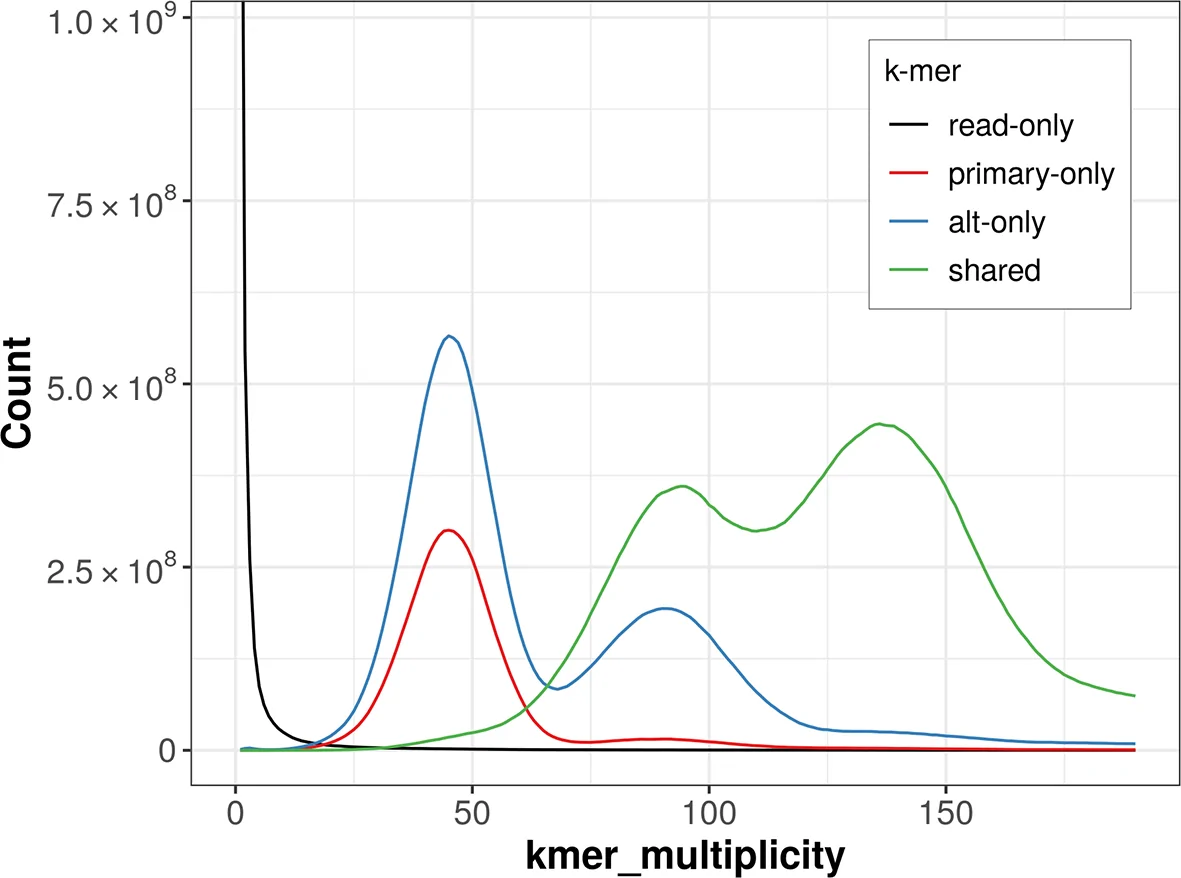
Evaluation of *k*-mer completeness using MerquryFK. This plot illustrates the recovery of *k*-mers from the original read data in the final assemblies. The horizontal axis represents *k*-mer multiplicity, and the vertical axis shows the number of *k*-mers. The black curve represents *k*-mers that appear in the reads but are not assembled. The green curve corresponds to *k*-mers shared by both haplotypes, and the red and blue curves show *k*-mers found only in one of the haplotypes.

The combined primary and alternate assemblies achieve an estimated QV of 60.8. The *k*-mer completeness is 57.07% for the primary assembly, 81.35% for the alternate haplotype, and 99.32% for the combined assemblies ( Figure 4).

**Figure 5.**
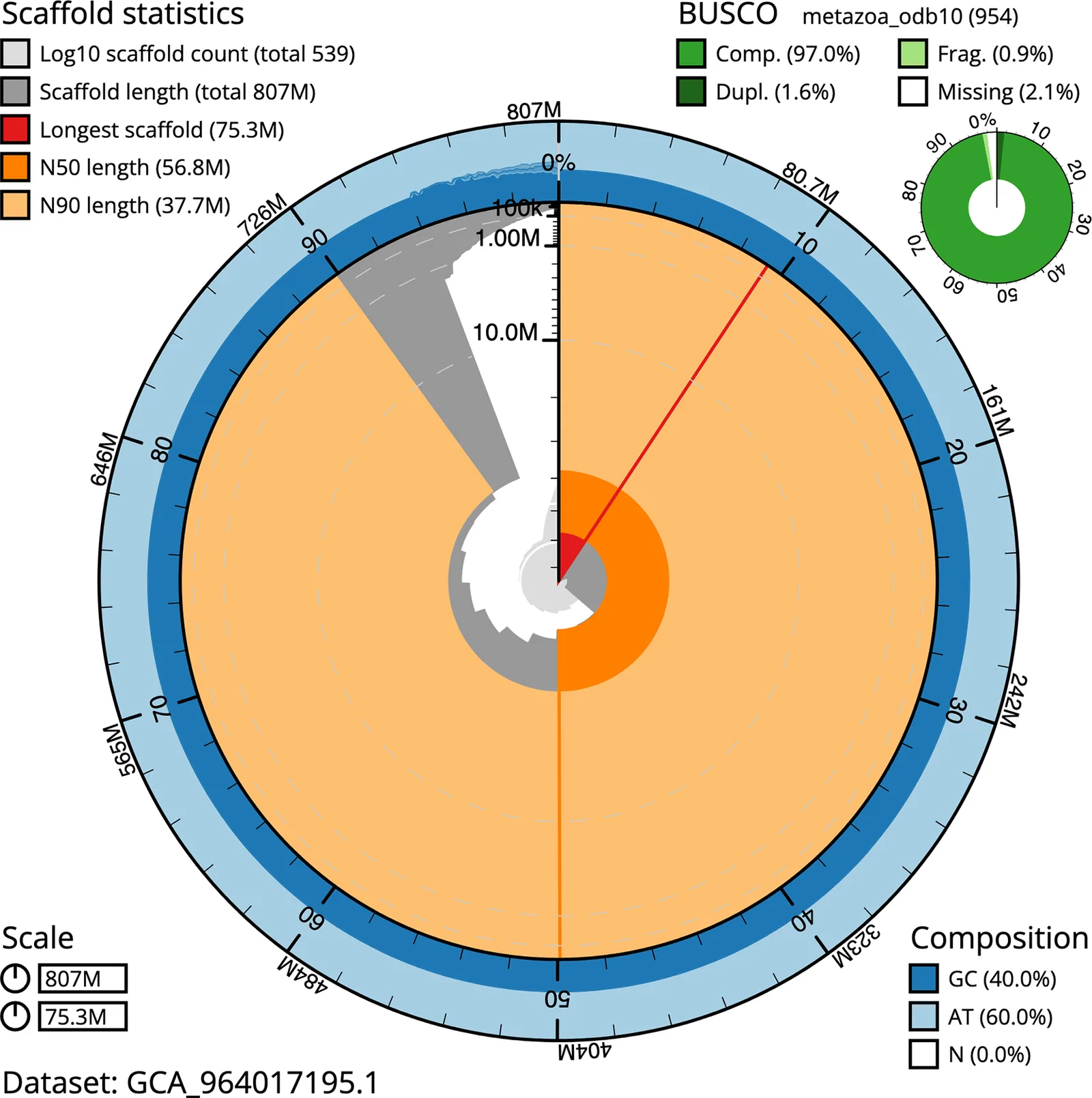
Assembly metrics for jaSidRadi1.1. The BlobToolKit snail plot provides an overview of assembly metrics and BUSCO gene completeness. The circumference represents the length of the whole genome sequence, and the main plot is divided into 1 000 bins around the circumference. The outermost blue tracks display the distribution of GC, AT, and N percentages across the bins. Scaffolds are arranged clockwise from longest to shortest and are depicted in dark grey. The longest scaffold is indicated by the red arc, and the deeper orange and pale orange arcs represent the N50 and N90 lengths. A light grey spiral at the centre shows the cumulative scaffold count on a logarithmic scale. A summary of complete, fragmented, duplicated, and missing BUSCO genes in the metazoa_odb10 set is presented at the top right. An interactive version of this figure can be accessed on the BlobToolKit viewer.

**Figure 6.**
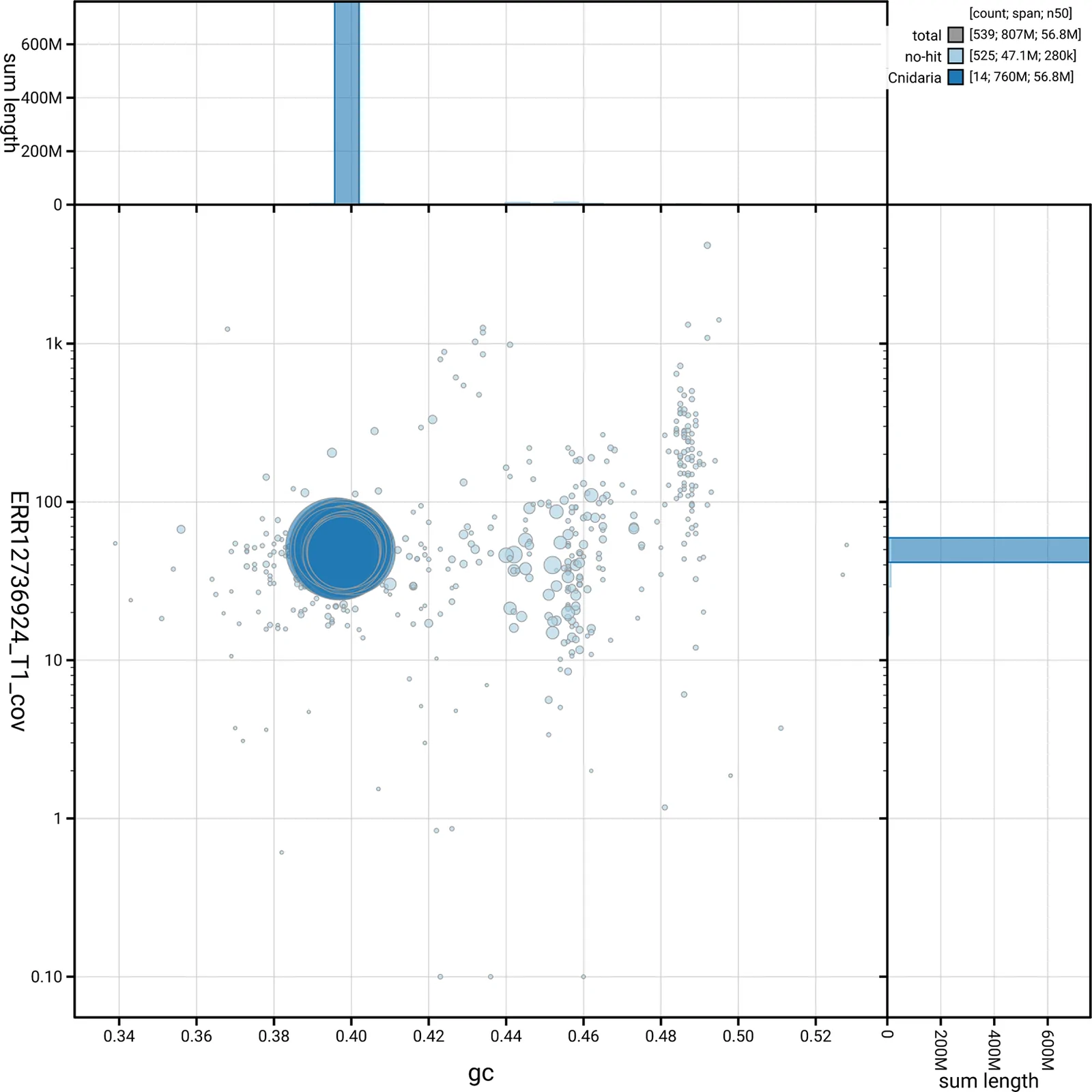
BlobToolKit blob plot for jaSidRadi1.1. The plot shows base coverage (vertical axis) and GC content (horizontal axis). The circles represent scaffolds, with the size proportional to scaffold length and the colour representing phylum membership. The histograms along the axes display the total length of sequences distributed across different levels of coverage and GC content. An interactive version of this figure is available on the BlobToolKit viewer.

BUSCO v.5.5.0 analysis using the metazoa_odb10 reference set ( *n* = 954) identified 97.0% of the expected gene set (single = 95.4%, duplicated = 1.6%). The snail plot in Figure 5 summarises the scaffold length distribution and other assembly statistics for the primary assembly. The blob plot in Figure 6 shows the distribution of scaffolds by GC proportion and coverage.

Table 4 lists the assembly metric benchmarks adapted from Rhie *et al.* (2021) and the Earth BioGenome Project Report on Assembly Standards January 2026. The EBP metric, calculated for primary assembly, is **6.C.Q63**, meeting the recommended 6.C.Q40 reference standard.

**Table 4.** Earth Biogenome Project summary metrics for the *Siderastrea radians* assembly.

Measure	Value	Benchmark
EBP summary (primary)	6.C.Q63	6.C.Q40
Contig N50 length	4.19 Mb	≥ 1 Mb
Scaffold N50 length	56.75 Mb	= chromosome N50
Consensus quality (QV)	Primary: 63.1; alternate: 60.0; combined: 60.8	≥ 40
*k*-mer completeness	Primary: 57.07%; alternate: 81.35%; combined: 99.32%	≥ 90%
BUSCO	C:97.0% [S:95.4%, D:1.6%], F:0.9%, M:2.1%, n:954	S > 90%; D < 5%
Percentage of assembly assigned to chromosomes	94.17%	≥ 90%

*Notes:* The EBP summary uses log10(Contig N50); chromosome-level (C) or log10(Scaffold N50); Q (Merqury QV). BUSCO: C = complete; S = single-copy; D = duplicated; F = fragmented; M = missing; n = orthologues.

### Genome annotation report

The *Siderastrea radians* genome assembly (GCA_964017195.1) was annotated by Ensembl at the European Bioinformatics Institute (EBI). This annotation includes 130 613 transcribed mRNAs from 47 051 protein-coding and 44 802 non-coding genes. The average transcript length is 9 235.47 bp, with an average of 1.42 coding transcripts per gene and 4.57 exons per transcript. Further details of this annotation are available from the Ensembl annotation page.

The *S. radians* genome GCA_964017195.1 was also annotated by Metz *et al*. (2026) using BRAKER3 v3.0.8 ( Gabriel *et al.*, 2024). The annotation comprises 45 387 coding sequences, with an average gene length of 1 211 bp, 1.13 coding transcripts per gene, and 4.99 exons per transcript. Annotation completeness was assessed using BUSCO v5.8.2 against the cnidaria_odb12 lineage dataset, yielding 95.6% complete (80.3% single-copy, 15.2% duplicated), 1.3% fragmented, and 3.1% missing BUSCOs. Annotation files are available from the GitHub repository ( Link).

## Metagenome report

**Figure 7.**
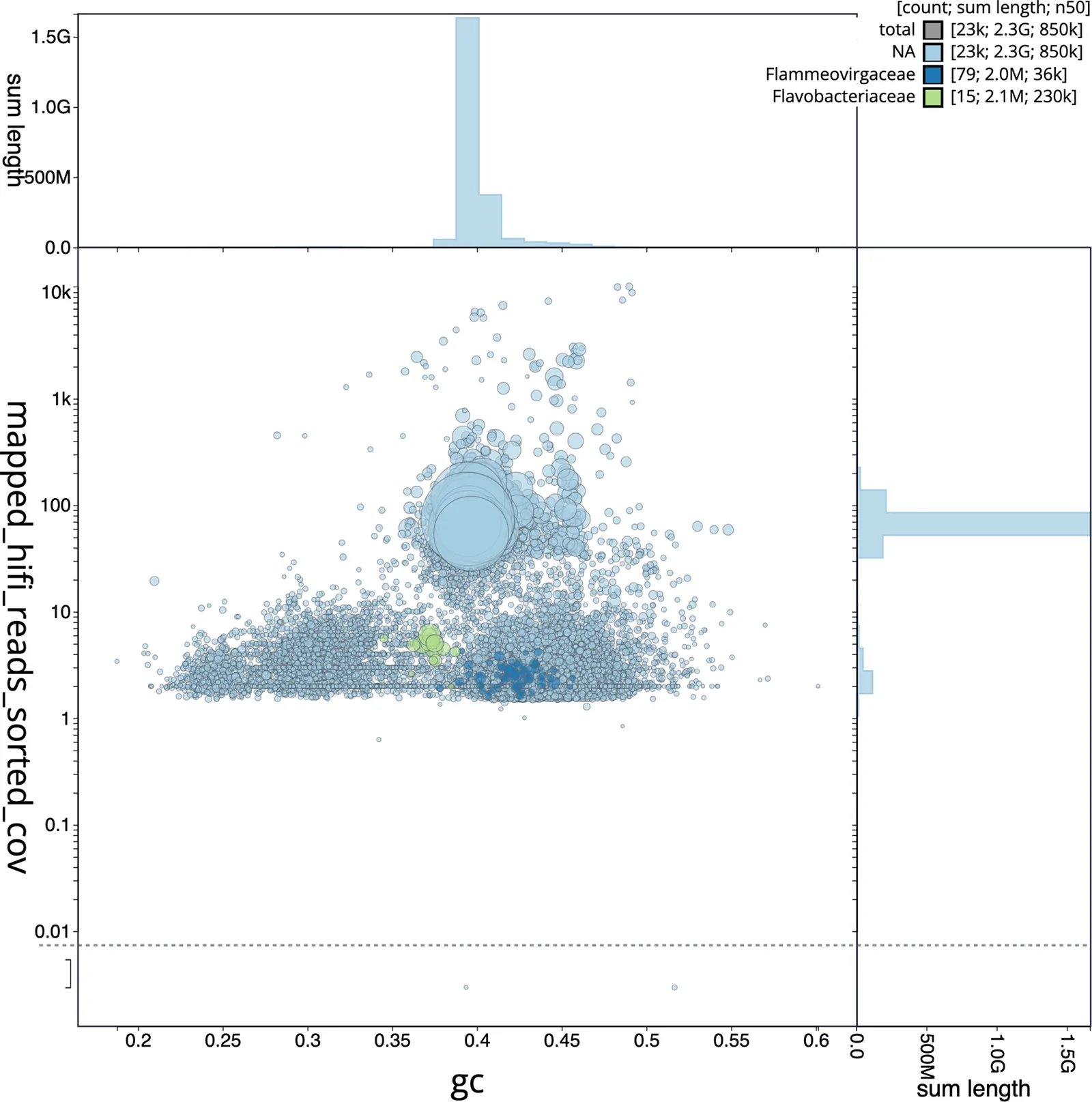
Blob plot for sequences in the *Siderastrea radians* metagenome. The plot shows base coverage (vertical axis) and GC content (horizontal axis). Binned contigs are coloured by family. Circles are sized in proportion to sequence length on a square-root scale. Histograms show the distribution of sequence length sum along each axis. An interactive version of this figure may be viewed here.

We recovered two binned metagenomes from the metagenome assembly ( Figure 7), neither of which met the criteria for MAGs. Both were assigned to the bacterial phylum Bacteroidota and class Bacteroidia, with genome sizes ranging from 2.05 to 2.12 Mbp (mean: 2.09 ± 0.03 Mbp). Mean completeness was 72.1% (± 11.8%) with 0.4% (± 0.4%) contamination. Table 5 summarises the taxa and quality of the metagenome bins.

**Table 5.** Quality metrics and taxonomic assignments of the binned metagenomes.

NCBI taxon	Taxid	GTDB taxonomy	Quality	Size (bp)	Contigs	Circular	Mean coverage	Completeness (%)	Contamination (%)
Flavobacteriaceae bacterium	1871037	Bacteroidia	MEDIUM	2120934	15	No	5.06	83.88	0.84
Flammeovirgaceae bacterium	2026740	Bacteroidia	MEDIUM	2053610	79	No	2.54	60.34	0

**Table 6.** Software versions and sources used for *Siderastrea radians.*

Software	Version	Source
BLAST	2.14.0	ftp://ftp.ncbi.nlm.nih.gov/blast/executables/blast+/
BlobToolKit	4.3.9	https://github.com/blobtoolkit/blobtoolkit
BUSCO	5.5.0	https://gitlab.com/ezlab/busco
bwa-mem2	2.2.1	https://github.com/bwa-mem2/bwa-mem2
CheckM	2015-01-16	https://ecogenomics.github.io/CheckM/
DAS Tool	1.1.2	https://github.com/cmks/DAS_Tool
DIAMOND	2.1.8	https://github.com/bbuchfink/diamond
dRep	3.4.0	https://github.com/MrOlm/drep
fasta_windows	0.2.4	https://github.com/tolkit/fasta_windows
FastK	1.1	https://github.com/thegenemyers/FASTK
GenomeScope2.0	2.0.1	https://github.com/tbenavi1/genomescope2.0
Gfastats	1.3.6	https://github.com/vgl-hub/gfastats
GTDB-Tk	1.2.1	https://github.com/Ecogenomics/GTDBTk
Hifiasm	0.19.8-r603	https://github.com/chhylp123/hifiasm
HiGlass	1.13.4	https://github.com/higlass/higlass
matplotlib	3.10.3	https://matplotlib.org/
MerquryFK	1.1.0-c1	https://github.com/thegenemyers/MERQURY.FK
MetaBAT2	2.15-15-gd6ea400	https://bitbucket.org/berkeleylab/metabat
MetaMDBG	Pre-release	https://github.com/GaetanBenoitDev/metaMDBG
Minimap2	2.24-r1122	https://github.com/lh3/minimap2
MitoHiFi	3	https://github.com/marcelauliano/MitoHiFi
MultiQC	1.14; 1.17 and 1.18	https://github.com/MultiQC/MultiQC
Nextflow	23.04.1; 23.10.0	https://github.com/nextflow-io/nextflow
PretextSnapshot	0.0.4	https://github.com/sanger-tol/PretextSnapshot
PretextView	1.0.3	https://github.com/sanger-tol/PretextView
Prokka	1.14.5	https://github.com/tseemann/prokka
purge_dups	1.2.3	https://github.com/dfguan/purge_dups
samtools	1.16.1; 1.17; 1.18; 1.6	https://github.com/samtools/samtools
sanger-tol/ascc	0.1.0	https://github.com/sanger-tol/ascc
sanger-tol/blobtoolkit	0.4.0	https://github.com/sanger-tol/blobtoolkit
sanger-tol/curationpretext	1.4.2	https://github.com/sanger-tol/curationpretext
Seqtk	1.4-r122	https://github.com/lh3/seqtk
Singularity	3.9.0	https://github.com/sylabs/singularity
TreeVal	1.0.2	https://github.com/sanger-tol/treeval
YaHS	1.1a.2	https://github.com/c-zhou/yahs

## Author information

Contributors are listed at the following links:

• Members of the Wellcome Sanger Institute Tree of Life Management, Samples and Laboratory team
• Members of Wellcome Sanger Institute Scientific Operations – Sequencing Operations
• Members of the Wellcome Sanger Institute Tree of Life Core Informatics team
• Members of the EBI Aquatic Symbiosis Genomics Data Portal Team
• The Aquatic Symbiosis Genomics Project leadership

## Wellcome Sanger Institute – Legal and Governance

The materials that have contributed to this genome note have been supplied by a Tree of Life collaborator. The Wellcome Sanger Institute employs a process whereby due diligence is carried out proportionate to the nature of the materials themselves, and the circumstances under which they have been/are to be collected and provided for use. The purpose of this is to address and mitigate any potential legal and/or ethical implications of receipt and use of the materials as part of the research project, and to ensure that in doing so we align with best practice wherever possible.

The overarching areas of consideration are:

• Ethical review of provenance and sourcing of the material
• Legality of collection, transfer and use (national and international)

Each transfer of samples is undertaken according to a Research Collaboration Agreement or Material Transfer Agreement entered into by the Tree of Life collaborator, Genome Research Limited (operating as the Wellcome Sanger Institute) and in some circumstances other Tree of Life collaborators.

## Data Availability

European Nucleotide Archive: Siderastrea radians (lesser starlet coral). Accession number PRJEB73731; BioProject URL: https://identifiers.org/ena.embl/PRJEB73731. The genome sequence is released openly for reuse. The *Siderastrea radians* genome sequencing initiative is part of the Aquatic Symbiosis Genomics Project (PRJEB43743) and the Sanger Institute Tree of Life Programme (PRJEB43745). All raw sequence data and the assembly have been deposited in INSDC databases. Raw data and assembly accession identifiers are reported in Tables 1 and 2. Production code used in genome assembly at the WSI Tree of Life is available at https://github.com/sanger-tol . Table 6 lists software versions used in this study.
