# Supplementary material for: The chromosomal genome sequence of the lesser starlet coral, Siderastrea radians (Pallas, 1766) (Scleractinia: Rhizangiidae) and its associated microbial metagenome sequences
Source: Wellcome Open Res. 2026 Jul 30;11:493. [Version 1] doi: 10.12688/wellcomeopenres.27286.1 (PMC13560801; doi:10.12688/wellcomeopenres.27286.1)
Supplement: Peer review history

# Reviewer report: Reviewer response for version 1

**DOI:** 10.21956/wellcomeopenres.30031.r163126

**Published:** 2026-08-21

**Authors:** Duong Huy Nguyen

**Affiliations:** 1 Vietnam Academy of Science and Technology, Hanoi, Vietnam

**Abstract and Background:** The background section explicitly states that the coral *Siderastrea radians* typically hosts the algal symbiont *Breviolum* (B5a), which highlights the complex holobiont structure of the organism. However, mentioning this detail inadvertently sets a strong expectation for the reader that the genomic data of the symbiotic algae will be analyzed or at least discussed in the subsequent sections. Since the actual results completely omit any data regarding this symbiont, it creates an internal contradiction within the manuscript. The authors need to refine the narrative to address this discrepancy and ensure logical consistency from the introduction to the results.

**Methods:** Regarding the nucleic acid extraction protocols, the authors should add a brief discussion clarifying the limitations of the mechanical tissue disruption method used. Specifically, the use of the automated cryo-pulverizer might not generate sufficient mechanical force to lyse the highly rigid cell walls of the symbiotic microalgae. Clarifying whether this extraction protocol was inherently biased toward successfully recovering primarily the host animal's DNA would provide a reasonable scientific basis to explain the unexpected absence of the algal symbiont genomic data in the downstream metagenomic analyses.

**Genome sequence report and Assembly metrics**: There is a significant discrepancy between the estimated genome size generated by GenomeScope2 (1,144.10 Mb) and the final assembly length (807.19 Mb), indicating that nearly 30% of the expected sequence length is unaccounted for. From a bioinformatics perspective, this is likely due to the high repeat content (77.42%) causing algorithms to collapse complex repetitive regions, or an initially inflated estimation caused by exogenous sequence noise. Regardless of the actual cause, the authors must include a transparent discussion explaining this size reduction to prevent confusion among readers.

 Additionally, the k-mer completeness for the primary assembly is reported at only 57.07%, which could easily mislead non-specialist readers into thinking the reference genome is severely incomplete. The authors must explicitly state in the text that this low percentage is actually a byproduct of highly efficient haplotype phasing by the assembly algorithm, which accurately pushed a large portion of heterozygous variants into the alternate haplotype, ultimately resulting in an excellent combined completeness of 99.32%. Adding a brief explanation will prevent any unwarranted doubts regarding the structural integrity of the primary assembly.

**Genome annotation report:** The gene prediction metrics present an unusually high number of protein-coding genes for a metazoan in the order Scleractinia. Both the Ensembl and BRAKER3 annotation pipelines reported over 45,000 to 47,000 protein-coding genes, whereas the typical functional gene count for coral species generally ranges between 20,000 and 35,000. This substantial surplus strongly indicates a high risk of over-annotation, which frequently occurs when transposable elements are not completely masked and are misidentified as true genes, or when excessively long genes are fragmented by the algorithm into multiple independent loci. The research team must add a discussion paragraph addressing this anomaly and explicitly advise future users to rigorously filter the dataset using functional assessment tools before utilizing it for downstream evolutionary or comparative genomic analyses.

**Metagenome report**: This section contains a significant ecological data gap that directly contradicts the biological context established in the introduction. As the authors affirmed that *Siderastrea radians* naturally hosts *Breviolum* microalgae, standard molecular biology principles dictate that whole-tissue extraction of a holobiont should yield a massive amount of algal genomic sequencing data. However, the reported metagenomic bins only identify two bacterial taxa assigned to Bacteroidota, completely omitting any mention or explanation regarding the disappearance of the symbiotic microalgae. This illogical absence must be transparently clarified. The authors should confirm whether the collected coral specimen was undergoing a bleaching event at the time of sampling, naturally leading to a loss of symbionts. Alternatively, if the algal genomic reads were successfully filtered out and reserved to be published independently as a separate project, this information must be explicitly stated in the manuscript to maintain scientific rigor and logical coherence.

 Overall, I consider this manuscript to be of interest to the scientific community and recommend its acceptance in Wellcome Open Research once these minor issues have been addressed.

Are sufficient details of methods and materials provided to allow replication by others?

Partly

Is the rationale for creating the dataset(s) clearly described?

Yes

Are the datasets clearly presented in a useable and accessible format?

Yes

Are the protocols appropriate and is the work technically sound?

Yes

Reviewer Expertise:

Bioinformatics, Genomics

I confirm that I have read this submission and believe that I have an appropriate level of expertise to confirm that it is of an acceptable scientific standard.

# Reviewer report: Reviewer response for version 1

**DOI:** 10.21956/wellcomeopenres.30031.r163120

**Published:** 2026-08-07

**Authors:** Nuria P Torres-Aguila

**Affiliations:** 1 Universitat de Barcelona, Barcelona, Spain

The Data Note submitted by C. Avelino et al. presents a newly assembled genome of the coral species *Siderastrea radians*, along with insights into its associated microbiome. The manuscript is well structured and provides sufficient detail regarding the genome assembly. Nonetheless, I have identified few minor issues:

- **Methods **section, *Sample acquisition*: “The jaSidRadi1 specimen was also used for RNA sequencing.”. I think this sentence is unnecessary, as this is described below. However, if the authors wish to clarify this here, I suggest adding that samples from this specimen were also used for genomic DNA extraction and the Hi-C procedure.
- In the PDF version, figures and tables appear before they are first cited in the text, making the article difficult to follow. Please review this.
- **Genome sequence report** section, *Assembly statistics*: “The scaffold count follows the NCBI assembly statistics and … where present.” I suggest moving this sentence to the Methods section (Genome assembly) or placing it in the same paragraph as the preceding sentence.
- **Genome sequence report** section, *Assembly quality metrics*: “The k-mer completeness is 57.07% for the primary assembly, 81.35% for the alternate haplotype, and 99.32% for the combined assemblies (Figure 4).” The markedly lower k-mer completeness of the primary assembly compared with the alternate assembly is unexpected. Please provide an explanation for this result and clarify whether it reflects the assembly strategy or post-processing steps rather than a quality issue of the assembly.
- **Genome sequence report** section, *Genome annotation report*: It is unclear whether the generated RNA-seq data were used for annotation. Including statistics related to the annotation process (e.g., the percentage of RNA-seq reads aligned to the assembly) would provide additional support for the quality of the annotation and indirect evidence for the completeness of the genome assembly.

Overall, I consider this manuscript to be of interest to the scientific community and recommend its acceptance in Wellcome Open Research once these minor issues have been addressed.

Are sufficient details of methods and materials provided to allow replication by others?

Yes

Is the rationale for creating the dataset(s) clearly described?

Yes

Are the datasets clearly presented in a useable and accessible format?

Yes

Are the protocols appropriate and is the work technically sound?

Yes

Reviewer Expertise:

Genomics, Transcriptomics, Developmental Genetics, Evolutionary Biology.

I confirm that I have read this submission and believe that I have an appropriate level of expertise to confirm that it is of an acceptable scientific standard.
